# Clinical features and pulmonary infections of diffuse alveolar hemorrhage in rheumatic disease patients

**DOI:** 10.1371/journal.pone.0339183

**Published:** 2025-12-18

**Authors:** Carla Marina Román-Montes, Eduardo Briones-García, Shaul Ariel Navarro-Lara, Marco Antonio Ortiz-Bustamante, Iris Paola García-Herrera, Guillermo Arturo Guaracha-Basañez

**Affiliations:** 1 Infectious Diseases Department, Instituto Nacional de Ciencias Médicas y Nutrición Salvador Zubirán, Mexico City, Mexico; 2 Immunology and Rheumatology Department, Instituto Nacional de Ciencias Médicas y Nutrición Salvador Zubirán, Mexico City, Mexico; Sutter Gould Medical Foundation, UNITED STATES OF AMERICA

## Abstract

**Introduction:**

Diffuse alveolar hemorrhage (DAH) is an uncommon but potentially life-threatening pulmonary complication in patients with rheumatic diseases (RD). Immunosuppression, endothelial injury, and systemic inflammation may increase the risk of pulmonary infections, further complicating clinical management and worsening outcomes.

**Objective:**

To describe the prevalence and factors associated with pulmonary infections in patients with DAH and RD.

**Methods:**

We conducted a retrospective, cross-sectional study including patients diagnosed with DAH and underlying RD at a tertiary care center in Mexico between 2003 and 2023. A logistic regression model was used to identify factors associated with infection.

**Results:**

Seventy-three patients with DAH were included; 31.5% had concurrent pulmonary infection. Systemic lupus erythematosus (68.5%) and ANCA-associated vasculitis (29%) were the most common underlying diseases. Evidence of hemorrhage in bronchoalveolar lavage was significantly more frequent in the infection group (76% vs. 48%; p = 0.02). Bacterial infection was the most common, occurring in 62% of cases, followed by fungal infection at 21%. The 30-day mortality rate was 10%, and infection was not an independent predictor of death. A multivariable logistic regression analysis revealed that male sex (OR 9.8; 95% CI: 2.7–35.3; p < 0.01) and IMV requirement (OR 8.8; 95% CI: 1.7–46.9; p = 0.01) were significantly associated with pulmonary infection.

**Conclusion:**

Pulmonary infections are common in patients with DAH and RD. A relatively low short-term mortality rate was observed in this cohort. Male sex and IMV requirement were associated with infections. Early microbiological assessment and targeted therapy are crucial to optimizing outcomes in this vulnerable population.

## Introduction

Diffuse alveolar hemorrhage (DAH) is a rare, life-threatening complication of rheumatic diseases (RD), particularly ANCA-associated vasculitis, anti-GBM disease, and systemic lupus erythematosus (SLE) [1]. Bleeding into alveoli causes respiratory distress and impaired gas exchange [2]. The prevalence of DAH in RD varies, but in SLE, the prevalence of DAH ranges from 0.6% to 5.4%.

DAH in RD results from autoimmune vascular injury and coagulopathy [3] and signals severe disease [4]. Main symptoms are hemoptysis, anemia, lung infiltrates, and acute respiratory failure. In-hospital mortality is consistently reported to be high, up to 50%, with no significant variations across different study periods and different RD [5–8]. DAH increases the risk of pulmonary infection, partly due to immunosuppression [9], which raises the likelihood of bacterial, fungal, viral, or parasitic infections and complicates management. Additionally, immune system changes due to ongoing inflammation and potential airway compromise can promote pathogen colonization, raising concerns about opportunistic infections in this population. This diversity necessitates different empirical treatments and may affect prognosis [10].

Pulmonary infection rates in DAH with RD are influenced by age, comorbidities, and disease severity [6,11,12]. Infected patients have more severe symptoms, longer hospital stays, and a higher need for Intensive Care Unit (ICU) or invasive mechanical ventilation (IMV) [13].

The study aimed to describe the prevalence and factors associated with pulmonary infections of DAH related to RD.

## Methods

We conducted a retrospective, cross-sectional, single-center study. We included all adult patients (≥18 years old) with DAH related to RD who were admitted to a tertiary care hospital in Mexico City between January 1, 2003, and December 31, 2023. Patients were identified from the institutional clinical file records.

### Data collection

Data collection began on September 10, 2024, and was completed on January 30, 2025. The main outcome was the presence of pulmonary infection to assess its prevalence. We collected demographic and clinical data, microbiological results, ICU admission and IMV needs, and 30-day mortality rates. The anonymity and confidentiality of data were preserved.

### Definitions

An episode of DAH was defined as 1) the presence of symptoms such as dyspnea, hemoptysis, or cough; 2) radiographic evidence of new ground-glass opacities or alveolar infiltrates on chest CT, or bronchoscopy confirmation of hemorrhage, indicated by persistent or increasing bloody return during bronchoalveolar lavage (BAL), cytopathologic evidence such as hemosiderin-laden macrophages, or histological confirmation, when available, characterized by pulmonary capillaritis; and 3) a decrease of ≥2 g/dL in hemoglobin without bleeding elsewhere.

A standardized definition of pulmonary infection, or pneumonia in DAH, does not exist. Pulmonary infection during the DAH episode was defined as a documented positive culture, molecular, or antigen test from lower airway samples or blood cultures within 48–72 hours of DAH admission. Further invasive fungal disease (IFI) was defined according to the criteria of the European Organization for Research and Treatment of Cancer (EORTC) and the Mycoses Study Group Education and Research Consortium (MSGERC) [14,15]. Galactomannan antigen (Platelia *Aspergillus* Ag Assay, ©Bio-Rad Laboratories, Inc.) has been available since 2013, and PCR testing (RespiFinder®22) for detecting pathogens causing pneumonia has been available since 2015. The RD activity was assessed by two independent rheumatologists using information from clinical chart notes. No disease-specific activity scales were employed; the evaluation relied solely on clinical and laboratory features.

### Statistical analysis

A descriptive analysis was conducted on the clinical characteristics of patients, utilizing categorical variables to display counts and proportions. Based on the data distribution (as determined by the Shapiro-Wilk W test), the data were presented as either mean and standard deviation (SD) or median and interquartile range (IQR). Comparisons between DAH with infection and DAH without infection, ICU versus non-ICU, and mortality were conducted using Chi-squared or Fisher’s exact tests and Student’s t-test or Wilcoxon Rank Sum test. Variables with a p-value less than 0.2 in the bivariate analysis and those showing biological plausibility were included in a stepwise multiple logistic regression to identify factors associated with pulmonary infections. The 95% confidence intervals (95% CI) are reported, and a p-value of less than 0.05 was considered statistically significant. All analyses were performed with STATA 14. (©StataCorp LLC, College Station, Texas, USA).

### Ethics statements

The Local Institutional Review Board approved the study (Reference number INF-4589). Due to its retrospective nature, written informed consent was waived.

## Results

### Baseline characteristics

A total of 73 patients with DAH were included. The median age of the cohort was 30 years (IQR, 23–39 years). Most patients were female (71%). Comorbidities were present in 34 (47%) patients; the most frequent were hypertension, chronic kidney disease, and diabetes. SLE was the most common underlying RD in 68.5% (n = 50), followed by ANCA-associated vasculitis in 29% (n = 21), rheumatoid arthritis in 1.25% (n = 1), and primary antiphospholipid syndrome in 1.25% (n = 1). The median RD duration was 2 years (IQR 0–6). The baseline clinical and treatment characteristics of the cohort are summarized in Table 1.

**Table 1:** Demographic and rheumatic disease characteristics in DAH.

	Overall n = 73 (%)	DAH Pulmonary infection n = 23 (%)	DAH without infection n = 50 (%)	*p*-value
Females	52 (71)	10 (43.5)	42 (84)	<0.01
Age, years*	30 (23–39)	29 (21-38)	30 (24–40)	0.75
Comorbidities	34 (47)	15(65)	19 (38)	0.03
Chronic kidney disease	17 (23)	8 (35)	9 (18)	0.11
Hypertension	14 (19)	8 (35)	7 (14)	0.04
Type 2 diabetes	4 (5.5)	3 (12)	1 (2)	0.11
Evolution of RD, years*	2 (0-6)	1 (0-5)	3 (0-8)	0.30
Activity of RD	31 (42.5)	8 (35)	24 (48)	0.75
SLE	50 (68.5)	16 (70)	34 (68)	0.89
Vasculitis ANCA	21 (29)	7 (30)	14 (28)	0.32
Immunosuppressant	34 (47)	12(52)	22(44)	0.51
Glucocorticoids	24 (33)	9 (39)	15 (30)	0.44
Prednisone dose, mg*	20 (10-30)	20 (15–30)	12.5(10-35)	0.54
Antimalarials use	35 (48)	11 (48)	24 (48)	1.0

Values with an asterisk (*) are described as the median with interquartile range (IQR). Abbreviations: Hb, hemoglobin; DAH, Diffuse alveolar hemorrhage; SLE, systemic lupus erythematosus; BAL, bronchoalveolar lavage; ICU, intensive care unit; IMV, invasive mechanical ventilation. Leukocytosis >10,500 cells/μL.

**Fig 1:**
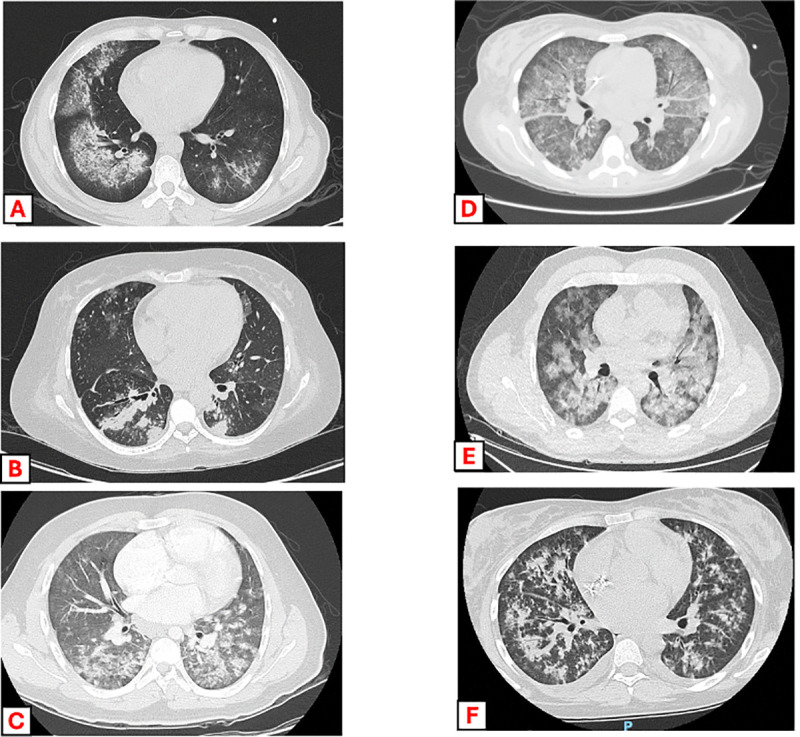
Chest CT findings in patients with diffuse alveolar hemorrhage, with and without pulmonary infections. (A–C) Cases with pulmonary infection: chest CT scans demonstrate bilateral ground-glass opacities and consolidations with superimposed interlobular septal thickening, patchy alveolar infiltrates, and areas of consolidation predominantly in the lower lobes, consistent with alveolar hemorrhage associated with coinfection. (D–F) Cases without pulmonary infection: chest CT scans reveal extensive bilateral ground-glass opacities and diffuse alveolar consolidation with a “crazy-paving” pattern, but without focal findings suggestive of superimposed infection.

Regarding DAH temporality, 40% of episodes were diagnosed before 2015. All patients underwent chest imaging compatible with DAH, including a chest X-ray in 99% and a chest CT in 83% of cases. Diffuse lung infiltrates were present in all chest X-rays. Bronchoscopy with BAL was performed in 63% (n = 46) of patients. Blood in tracheal aspiration was documented in 22% (11/50), and cytopathological evidence of hemorrhage was observed in 41% (n = 14). Overall, 7 (10%) patients died within 30 days of the DAH episode. The clinical and radiologic features of DAH are presented in Table 2 and illustrated in Fig 1.

**Table 2:** Clinical features of DAH presentation.

	Overall n = 73 (%)	DAH Pulmonary infection n = 23 (%)	DAH without infection n = 50 (%)	*p*-value
Dyspnea	73 (100)	23 (100)	50(100)	–
Hb 2 gr/dL decrease	45(62)	14(61)	31(62)	0.92
Hemoptysis	57 (78)	19 (83)	38(76)	0.52
Blood in BAL	46(63)	19 (83)	27 (54)	**0.02**
Leukocytes,10^3^/μL*	8.5 (4.9-11.9)	7.0 (4.6-11.3)	9.4(5.7-12.4)	0.26
Hemoglobin, g/dL*	9 (7.9-10.5)	9.1(8.3-10.4)	8.7(7.7-10.5)	0.37
Platelet, 10^3^/μL *	171(109-2069)	188(64-215)	167(115-271)	0.70
Leukocytosis	31 (42.5)	8(35)	23(46)	0.36
Lymphopenia	49 (67)	18(78)	31(62)	0.17
Previous antibiotic	19 (28)	5/21(24)	14/46(30)	0.57
Outcomes				
Septic Shock	7(10)	2(13)	4(8)	0.67
ICU admission	50(68.50)	19(83)	31(62)	0.10
Invasive mechanical ventilation	50 (68.5)	20(87)	30(60)	0.03
Days stay length*	15 (8-26)	17(10-32)	15(8–22)	0.15
30-day mortality	7(10)	4(17)	3(6)	0.19

Values with an asterisk (*) are described as the median with interquartile range (IQR). Abbreviations: Hb, hemoglobin; DAH, Diffuse alveolar hemorrhage; SLE, systemic lupus erythematosus; BAL, bronchoalveolar lavage; ICU, intensive care unit; IMV, invasive mechanical ventilation. Leukocytosis >10,500 cells/μL.

### Comparisons according to pulmonary infection

Pulmonary infection occurred in 23 (31.5%) patients. When patients with and without infection were compared, there were no significant differences in age [29 (IQR 21–38) vs. 30 (IQR 24–40), p = 0.75] or RD duration (p = 0.30). Females were less frequent in the infection group (19% vs. 81% of females, p < 0.01). Comorbidities were more common among patients with infection (65% vs. 38%, p = 0.03); the frequencies of hypertension (32% vs. 12.5%, p = 0.04), chronic kidney disease (28% vs. 20%, p = 0.49), and diabetes (12% vs. 2%, p = 0.11) were higher in this group.

The median prednisone dose was similar between patients with and without infection [20 mg (15–30) vs. 12.5 mg (10–35), p = 0.54]. Patterns of immunosuppressant use did not differ significantly: mycophenolate (25% vs. 18%), cyclophosphamide (0% vs. 27%), azathioprine (25% vs. 9%), and rituximab (8% vs. 0%) (all p > 0.05). Disease activity other than DAH was comparable (43.5% vs. 48%, p = 0.72), with renal involvement as the most frequent active site (40% vs. 35%, p = 0.79).

DAH-related characteristics were also similar between groups in terms of calendar period: 39% vs. 42% of DAH episodes occurred before 2015 (p = 0.82). Abnormal chest CT findings were present in a similar proportion of patients with and without infection (76% vs. 83%, p = 0.48), with comparable frequencies of micronodules (50% vs. 45%, p = 0.77), nodules (12.5% vs. 20%, p = 0.70), and consolidations (19% vs. 15%, p = 0.70). Patients with infection were more likely to undergo bronchoscopy with BAL (82% vs. 54%, p = 0.02) and to have visible blood during bronchoscopy (83% vs. 54%, p = 0.02). Cytopathological evidence of hemorrhage was more frequent in the infection group (64% vs. 26%, p = 0.04), whereas blood in tracheal aspirate was less commonly documented (10% vs. 30%, p = 0.16). Thirty-day mortality rates were 16% in patients with infection and 6% in those without infection (p = 0.22).

### Comparisons between SLE and ANCA-associated vasculitis

When comparing RD subsets, SLE was more frequent among women than ANCA-associated vasculitis (78% vs. 11%, p = 0.03) and occurred at a younger age [median 26.5 years (IQR 21–34) vs. 44 years (IQR 28–54), p < 0.01]. SLE patients had lower rates of overall disease activity at the time of DAH (34% vs. 67%, p = 0.01). The proportions of patients receiving immunosuppressants (50% vs. 43%, p = 0.58), the prevalence of pulmonary infection (32% vs. 33%, p = 0.91), and 30-day mortality (12% vs. 5%, p = 0.66) were similar between SLE and ANCA-associated vasculitis.

### Pulmonary infections

**Fig 2:**
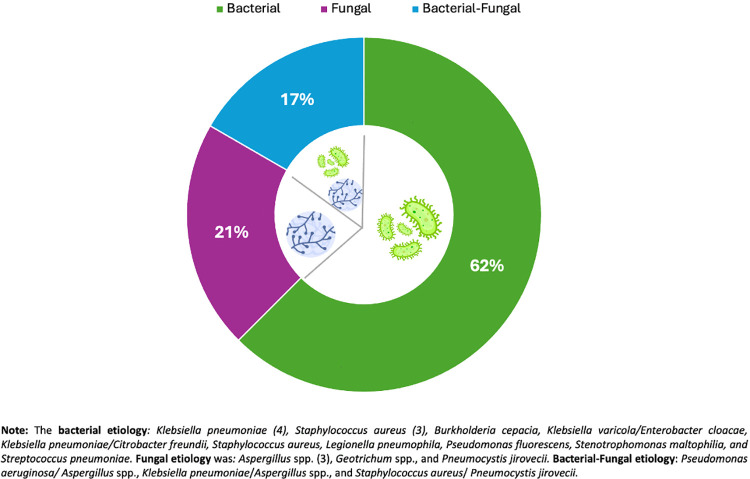
Etiology and different isolates in DAH with pulmonary infection.

Among the 23 patients with pulmonary infection, different diagnostic approaches were used to obtain microbiological evidence. Nine (39%) cases were diagnosed by BAL culture, six (26%) by tracheal aspirate culture, and two (9%) by both BAL and tracheal aspirate cultures. Three patients (13%) had positive blood cultures, two (9%) had a positive galactomannan antigen test, and two (9%) had both BAL culture and galactomannan positivity; one patient (4.5%) had a positive pneumococcal urinary antigen test. Bacteria were the most common cause of pulmonary infection; the microbiological etiology is shown in Fig 2. The multivariable logistic regression analysis of factors associated with pulmonary infection is presented in Table 3.

**Table 3:** Multivariable Logistic Regression Analysis of Factors Associated with infection.

	Odds Ratio (OR)	95% CI	*p-*value
Male sex	9.8	2.7-35.3	<0.01
Invasive mechanical ventilation	8.8	1.7-46.9	0.01
Systemic lupus erythematosus	2.4	0.5-10.7	0.23
Lymphopenia	2.8	0.7-11.3	0.14

Note: The overall model demonstrated good fit (LR χ² (4) = 25.21, p < 0.001) with a pseudo-R² of 0.28. The model’s specificity was 96%, and its sensitivity was 52%. Discrimination was acceptable, with an area under the ROC curve of 0.83.

## Discussion

This study provides valuable insight into a group of RD patients with DAH. The main finding shows a high rate of pulmonary infection. Additionally, we found that male sex and the need for IMV were independent factors significantly associated with the presence of infection.

The high rate of infection highlights the challenge of managing DAH in RD, as the clinical signs of sterile pulmonary capillaritis and infectious pneumonia are similar. Both can cause symptoms like fever, cough, hemoptysis, hypoxemia, and new alveolar infiltrates visible on imaging [16]. This diagnostic ambiguity presents a significant therapeutic challenge, as the primary treatment for DAH, an intense immunosuppressant, could be disastrous if given in the presence of an unrecognized infection. Our data strongly support a low threshold for bronchoscopy with BAL in all patients suspected of DAH. BAL is essential for confirming alveolar hemorrhage and, equally important, for collecting samples for detailed microbiological testing. The counterintuitive finding that macroscopic hemorrhage on BAL was more common in the infection group (76% vs. 48%) further emphasizes that the severity of hemorrhage cannot be used to rule out an infection. This aligns with studies suggesting that infection may trigger or worsen autoimmune-related DAH [17] or that pneumonia could lead to hemorrhage in the alveoli [10].

The factors associated with infection are very helpful for clinical risk evaluation. The strong link between IMV and ICU admission and infection probably indicates both severe disease and critical condition. This suggests that for any RD patient with DAH who worsens to the point of needing intensive care, infection should be considered the default assumption until proven otherwise. The association between male sex and pulmonary infection is a striking finding that merits further investigation [18]. While the reasons are likely multifactorial, several explanations can be postulated. Firstly, as SLE constituted most of our cohort and has a well-known female predominance, the smaller number of male SLE patients may represent a subset with more severe or atypical disease, potentially requiring more aggressive immunosuppression [18]. Secondly, there may be sex-based differences in comorbidities (e.g., smoking history) or social factors that influence infection risk. This association should be validated in larger, multi-center studies to explore its biological and epidemiological basis.

Pulmonary infection did not significantly affect the overall use of immunosuppressants, corticosteroids, or antimalarials; this differs from reports in patients with similar characteristics [12]. Median prednisone doses were similar, indicating that infection may be more related to comorbid burden or immune system dysregulation rather than the level of immunosuppression alone. However, the potential role of steroid-induced immunosuppression as a risk factor should not be overlooked, particularly in patients with additional vulnerabilities [19]. While immunosuppression and disease activity remain key factors in the development of DAH, comorbid conditions such as hypertension and diabetes may also increase the risk of superimposed infection [20].

The microbial spectrum observed in our study, dominated by bacterial and fungal pathogens, aligns with the expected profile of immunocompromised hosts [11,21]. The high rate of fungal infection is notable; these opportunistic infections, particularly *Pneumocystis jirovecii* and *Aspergillus fumigatus,* are well-recognized causes of acute respiratory compromise in patients receiving high-dose glucocorticoids or cytotoxic therapy. It should encourage clinicians in similar settings to perform early bronchoscopy and microbiological testing, including stains, fungal cultures, and antigen detection (e.g., galactomannan, β-D-glucan) [22]. This could support the empirical use of antifungals in certain high-risk patients while awaiting confirmatory results in clinical practice. The risk of these infections varies among RD; ANCA-associated vasculitis generally requires more intensive and longer immunosuppression, leading to a higher incidence of *Pneumocystis jirovecii* and other opportunistic pathogens compared to SLE cohorts. In contrast, patients with SLE can develop DAH early in their disease, often before significant cumulative immunosuppression, which may help explain the lower reported burden of opportunistic infection in this group. Our findings support this difference in risk, emphasizing the importance of being vigilant for opportunistic pathogens in ANCA-vasculitis–related DAH and considering pulmonary infection in the context of the underlying disease and treatment intensity.

The mortality rate observed was lower than that reported in previous studies, which have described rates as high as 42% in RD-related DAH. This difference could be due to variations in case severity, quicker access to tertiary care, or differences in diagnostic and treatment approaches, including the use of empiric antimicrobial therapy in many patients [22]. Notably, studies in the Mexican population have shown higher mortality rates, often linked to delayed diagnosis, advanced immunosuppression, or superimposed infections [23–25].

Our study has several limitations inherent to its retrospective, single-center design. The sample size, although significant for this rare condition, may limit the ability to identify additional risk factors or to fully assess the impact of infection on mortality. The specific patient population and microbiological environment of our tertiary care center in Mexico may affect the applicability of our findings to other settings. Additionally, diagnostic resources for detecting pulmonary infections improved significantly over the 20-year study period, with our center gradually adopting more sensitive microbiological, serological, and imaging tools. As a result, viral, atypical, or opportunistic infections may have been misdiagnosed in earlier years, and the overall prevalence reported here should be viewed as a conservative estimate. This limitation may have decreased our ability to identify infections. Finally, because there was no standardized protocol, treatment decisions regarding immunosuppression and antibiotics were left to the physicians’ discretion, which could introduce confounding factors. These findings underscore the complexity of mortality risk in DAH, suggesting that larger, multicenter studies may be necessary to better understand the independent prognostic significance of immunosuppression and other clinical factors.

## Conclusion

Pulmonary infection is a common complication in patients with RD presenting with DAH. Pulmonary infection should be actively identified through prompt bronchoscopy and BAL, especially in male patients and those requiring IMV and ICU admission. The relatively low mortality observed, unaffected by infection, supports a management approach that combines early, broad microbiological testing with immunosuppressive treatment. A high level of suspicion and a dual-treatment approach are essential for optimizing outcomes in these complex and vulnerable patients.
